# Utilizing response surface methodology for the microstructural examination of CeO_2_/GNP composite coating applied via air plasma spraying and post-treated with spark plasma sintering

**DOI:** 10.1016/j.heliyon.2024.e29411

**Published:** 2024-04-17

**Authors:** Ali Jounaki, Seyedeh Zahra Anvari

**Affiliations:** Department of Mechanical and Metallurgical Engineering, Payame Noor University, Tehran, Iran

**Keywords:** Air plasma spray: CeO_2_, GNP composite: spark plasma sintering: response surface methodology: porosity

## Abstract

CeO_2_/GNP coatings were fabricated using a three-step process: high-energy ball milling, spray-drying, and air plasma spray (APS) deposition. Spark plasma sintering (SPS) was employed for post-treatment to densify the coatings. Response surface methodology (RSM) was employed to optimize SPS processing parameters, including temperature, pressure, and holding time, for minimizing porosity. A quadratic model was developed and validated using analysis of variance (ANOVA) to determine the influence of the independent variables. A regression equation was derived to predict porosity based on the process parameters. Scanning elctron microscopy(SEM) measurements revealed a significant 16 % porosity reduction in the densified coating. Interlamellar cracks and microcracks were significantly reduced, improving coating integrity and interface bonding. Enhancement of SPS parameters indicated that higher temperature and pressure combined with shorter holding times yielded minimal porosity. The study demonstrated that higher pressures exhibited a stronger impact on porosity at higher temperatures, while the effect of temperature was more pronounced at a holding time of 4 min. The interaction between pressure and holding time highlighted the importance of considering both parameters for effective porosity control in SPS. The constraints for each parameter have been defined through the optimization process. The set temperature is 950 °C, the pressure is established at 30 MPA, and the duration of the holding time is 4 min.

## 1 Introduction

Advanced ceramic coatings with tailored microstructures and improved properties have gained significant attention in various industrial applications, including aerospace, automotive, and energy sectors. Among the promising coating materials, CeO_2_ (cerium oxide) and GNP (graphene nanoplatelets) have emerged as notable candidates due to their unique properties, such as high thermal stability, chemical inertness, and electrical conductivity [[1], [2], [3]]. Cerium Oxide deposited by Air Plasma Spray (APS) shows enhanced performance. It offers up to 15 times corrosion resistance in NaCl solution, 50 % improvement in hardness, and significant increases in elastic modulus (98 %) and fracture toughness (185 %) [4]. It also provides excellent wear-resistance due to its hydrophobicity and good thermal stability [[4], [5], [6]]. Recently, there has been a growing focus on ceramic composites that are strengthened with various forms of carbon nanomaterials. These include Carbon Nanotubes (CNTs), graphene, graphene oxide, carbon nanofibers, and graphene nanoplatelets. The increased attention is due to several enhanced properties of these composites. They exhibit improved mechanical strength, exceptional resistance to corrosion, and a remarkable ability to halt crack propagation [7,8]. Furthermore, they possess excellent thermal and electrical conductivity [9]. Graphene nanoplatelets, composed of stacked graphene sheets, have been identified as one of the most potent reinforcing agent among carbon-based nanofillers, thanks to their extraordinary ability to enhance material strength [10]. Their ultra-high aspect ratio and remarkably large specific surface area (2630 m^2^/g) enable them to form strong, intimate bonds with the matrix material, leading to significantly enhanced material properties [11,12]. However, these coatings also face certain challenges. One of the main issues is the presence of porosity in the coatings [13], which can result in reduced coating integrity, increased permeability to gases or liquids, and diminished mechanical properties. Porosity can be introduced during the plasma spray process due to the incomplete melting and solidification of the ceramic particles, leading to the formation of voids, cracks, and pores. Furthermore, during the coating deposition process, interfacial defects such as pores, cracks, or voids can form, which can result in cracking and delamination at the interface between the coating and substrate, thereby compromising adhesion. These issues require careful consideration and optimization of the plasma spray process parameters, material selection, and post-treatment techniques to mitigate the problems associated with plasma spray ceramic coatings and ensure their successful application in various industrial applications. Several studies have investigated the effects of various post-treatment methods on APS coatings, including Spark Plasma Sintering (SPS), hot pressing, annealing, and other heat treatment techniques [[14], [15], [16]].

SPS can help to reduce porosity by promoting further densification of the coating through localized heating and high-pressure consolidation [[16], [17], [18]].Additionally, SPS can induce controlled phase transformations of the ceramic material, improving its stability and performance with controlled nanostructure size and morphology [17]. In addition, SPS has the capability to improve the bond between the coating and the substrate [19].

The SPS process is influenced by various parameters such as temperature, pressure, and holding time, which can affect the microstructure and properties of the resulting material [20]. However, the selection of these process parameters can be challenging due to their complex interdependencies and the need for multiple experiments. This is where Response Surface Methodology (RSM) with a Box-Behnken design can be beneficial [21,22]. RSM allows for the systematic evaluation of process parameters at different levels, reducing the number of experiments required and providing a statistical model for predicting optimal process conditions. The Box-Behnken design, a type of RSM, can efficiently explore the parameter space with fewer experiments, saving time and resources. Thus, RSM with a Box-Behnken design can be a valuable approach to overcome the limitations of SPS process parameter optimization and enhance the efficiency of process development for obtaining desired material. To gain a comprehensive understanding of the microstructure and its relationship to processing parameters, this study investigated the microstructure of CeO_2_/GNP composite coatings fabricated using APS and subjected to SPS as post-treatment. RSM, a powerful tool for evaluating process parameters was employed throughout the investigation.CeO_2_ coatings have been chosen due to their potential in various applications such as catalyst and photocatalyst systems like fuel cells [23], corrosion inhibitors [24], biomedical uses due to low systemic toxicity [25], and enhanced wear resistance [26]. Stainless steel of various grades is commonly employed as a base for CeO_2_ coatings, which are applied using different processes. Past research has consistently shown the superior corrosion resistance of CeO_2_ coatings produced through a variety of techniques. The most common methods for producing CeO_2_ currently include electrodeposition [27,28], electron beam physical vapor deposition (EBPVD) [29], and chemical vapor deposition (CVD) [30].

The outcomes of this study are expected to improve the perception of the interrelated relationships between microstructure and properties in CeO_2_/GNP composite coatings. Additionally, valuable guidelines for tailoring SPS parameters to achieve enhanced coating performance will also be provided.

## 2 Materials and methods

### 2 .1Preparation

**Fig. 1:**
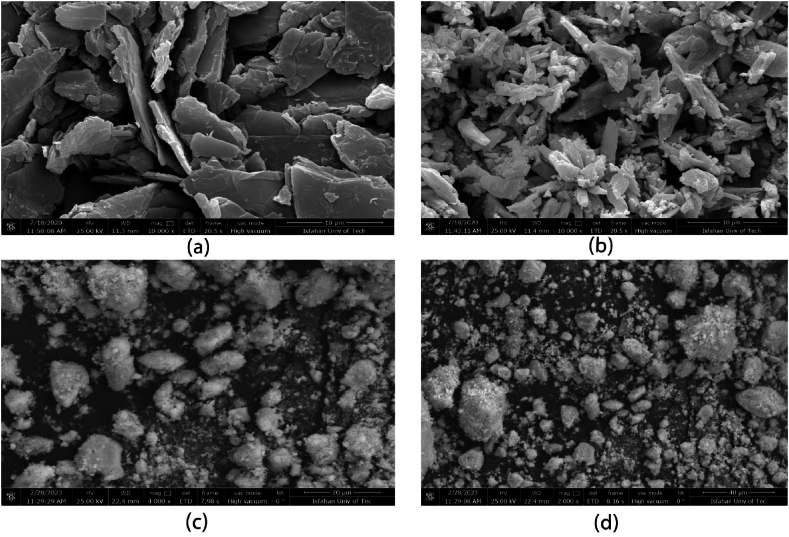
Field Emission Scanning Electron Microscopy (FE-SEM) images of (a) GNP powders (b) Ceria powders (c,d) agglomerated powders after spray drying.

Commercially available CeO_2_ with a particle size of <5 μm (Nanographi, USA) and graphene nanoplatelets (few layered) with a surface area of >110 m^2^/g (>97 % purity) were obtained from UGOX India. The FE-SEM (QUANTA FEG-450, FEI company, USA) images of graphene nanoplatelets and cerium oxide are represented in Fig. 1(a and b) respectively. To prevent mechanical fusion and achieve a pure powder mixture, a uniform blend of CeO_2_ with 2 % graphene nanoplatelets (GNP) was produced. The synthesis process was carried out using a high-energy planetary ball mill (Retesch PM 100). The milling was performed for a period of 2 h, utilizing stainless steel balls with a ball to powder ratio of 1:10. The mill operated at a speed of 300 RPM, and the entire process was conducted in an Argon atmosphere. Then the powders were sieved to be used for spray drying. The agglomerated micron-size powders were processed via spray drying. In this regard, the powders were dispersed in an aqueous organic binder to form a slurry, and the slurry was passed through the atomizer spray drying. Atomizer speed was kept at 9000 rpm at a constant temperature of 130 °C and a slurry with 350 g CeO_2_/GNP, 2 % polyvinyl alcohol (PVA), and 1250 g of water was formed. Fig. 1(c and d) displays the FE-SEM images of powders that have formed agglomerates. Additionally, the size distribution of the agglomerated powders was measured to be in the range of 20–80 μm. To have a better deposition efficiency in plasma spray the agglomerated powders were then sieved to form a standardized distribution of powders between 40 and 80 μm.

### 2.2 Manufacturing methods

In this study, a bond coat composed of NiCrAlY (Nickel Chromium Aluminum Yttrium), specifically the commercially available Amdry 962 from Oerlikon Metco, was utilized. The role of this bond coat is to enhance the adhesion between the substrate and the top coat. Application of the bond coat was achieved using an APS technique, resulting in a layer approximately 100 μm thick. The CeO_2_/GNP agglomerated powders were introduced into an APS jet, specifically a Metco 3 MB gun, following the optimized parameters outlined in Table 1. These powders were then deposited onto 16 stainless steel discs (grade 316), each with a diameter of 2.5 cm and a thickness of 0.5 cm. Ultimately, the As-APSed coatings underwent exposure to a SPS Model 10 Ton (Nanozint10) apparatus produced by KHPF Inc. The Box-Behnken matrix design of the RSM investigated three variables: temperature (at levels of 850, 900, and 950), pressure (at levels of 20, 25, and 30), and holding time (at levels of 5, 10, and 15). A total of 16 experimental runs with four center points to assure the accuracy of the experiment were carried out. Table 2 displays the specifics of each parameter along with their respective levels.

**Table 1:** The optimized factors of air plasma spray.

Plasma parameters	Value (unit)
Voltage	58 V
Current	515 A
Secondary gas	15 SCFH
Primary gas	80 SCFH
Powder feed rate	20 g/min
Stand of distance	80 mm

**Table 2:** Design matrix and experimental result of SPS process parameters with their corresponding level.

	Temperature (°C) **−1 0 + 1** **850 900 950**	Pressure (MPa) **−1 0 + 1** **20 25 30**	Holding time (Min) **−1 0 + 1** **5 10 15**	porosity level(%)
Run	A:A	B:B	C:C	R
1	900	25	10	9
2	850	25	5	10.5
3	950	20	10	7
4	850	20	10	6
5	900	30	15	7
6	900	20	15	4
7	900	25	10	9
8	850	25	15	5
9	950	30	10	7
10	950	25	5	8
11	900	20	5	9
12	850	30	10	8
13	950	25	15	7
14	900	25	10	9
15	900	30	5	8
16	900	25	10	8.8

### 2.3 Characterization

**Fig. 2:**
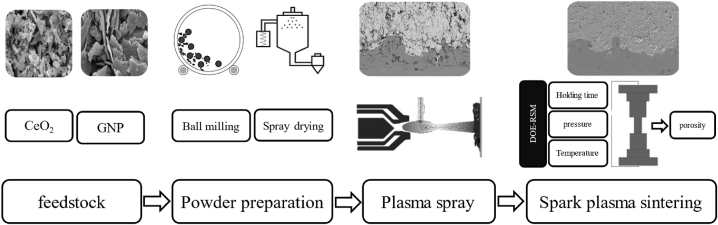
Schematic illustration of coating arrangement.

To study the microstructural evolution of the as-SPSed coatings, scanning electron microscope (SEM) (Philips XL30, FEI Ltd) images of the coating was provided. Fig. 2 illustrates the diagrammatic representation of the process, starting from the preparation of the powder, followed by APS, and concluding with SPS. In this study, the porosity of the final specimens was determined by analyzing SEM images taken at different magnifications:200 μm, 100 μm, and 50 μm, and based on ImageJ software. The average thickness of the coatings was determined by analyzing SEM images at a magnification of 200 μm. The mean thickness for each specimen is displayed in Table 3. The experimental design matrix with porosity percentage as a response is illustrated in Table 3.

**Table 3:** Microstructure evaluation and observation of densified coatings by SPS.

	Parameters	SEM image (100 μm)	Evaluation
1	T = 900 °C P = 25 MPa HT = 10 min		Porosity level = 9 % Mean Thickness = 163 μm
2	T = 850 °C P = 25 MPa HT = 5 min		Porosity level = 10.5 % Mean Thickness = 153 μm
3	T = 950 °C P = 20 MPa HT = 10 min		Porosity level = 7 % Mean Thickness = 175 μm
4	T = 850 °C P = 20 MPa HT = 10 min		Porosity level = 6 % Mean Thickness = 209 μm
5	T = 900 °C P = 30 MPa HT = 15 min		Porosity level = 7 % Mean Thickness = 176 μm
6	T = 900 °C P = 20 MPa HT = 15 min		Porosity level = 4 % Mean Thickness = 146 μm
7	T = 900 °C P = 25 MPa HT = 10 min		Porosity level = 9 % Mean Thickness = 205 μm
8	T = 850 °C P = 25 MPa HT = 15 min		Porosity level = 5 % Mean Thickness = 179 μm
9	T = 950 °C P = 30 MPa HT = 10min		Porosity level = 7 % Mean Thickness = 114 μm
10	T = 950 °C P = 25 MPa HT = 5 min		Porosity level = 8 % Mean Thickness = 168 μm
11	T = 900 °C P = 20 MPa HT = 5min		Porosity level = 9 % Mean Thickness = 213 μm
12	T = 850 °C P = 30 MPa HT = 10 min		Porosity level = 8 % Mean Thickness = 161 μm
13	T = 950 °C P = 25 MPa HT = 15min		Porosity level = 7 % Mean Thickness = 219 μm
14	T = 900 °C P = 25 MPa HT = 10 min		Porosity level = 9 % Mean Thickness = 159 μm
15	T = 900 °C P = 30 MPa HT = 5 min		Porosity level = 8 % Mean Thickness = 142 μm
16	T = 900 °C P = 25 MPa HT = 10min		Porosity level = 8.8 % Mean Thickness = 190 μm

### 2.4 Governing equations

In this section, the regression equation is presented, which has been derived from the RSM analysis. It provides a mathematical representation of the relationship between the response variable (porosity) and the independent variables (temperature, pressure, and holding time) in the experimental design. Porosity can be represented mathematically as a function of temperature (T), pressure (P), and holding time (HT), denoted as Porosity = f (T, P, HT). Table 3 displays the microstructural images corresponding to each run, showcasing the input variables employed in the study. Additionally, the thickness of each run is mentioned, providing important insights into the dimensional characteristics of the coatings. In this research, the empirical equation was developed using the Design Expert V 13.0.5 software. The equation incorporates the main and interaction effects of all factors, such as temperature, pressure, and holding time. It provides a mathematical representation of the relationship between these variables and the porosity response. The empirical equation serves as a valuable tool for predicting porosity levels under different process conditions, and it was obtained through statistical analysis to account for the complex interactions among the factors. Furthermore, the regression equation expressed in terms of coded equation (1) and the actual equation (2) enables making predictions about the response at specific levels of each factor. The coded and actual equations are provided below; A = temperature, B = pressure, C = holding time.

Coded equation:

(1) R = 9.649–0.7375 * A - 0.1 * B - 0.4605 * C - 0.5 * AB + 0.675 * AC + 0.6 * BC - 0.6625 * A^2^ - 1.2875 * B^2^ - 0.2385 * C^2^

The provided regression equation includes coefficients for factors A, B, and C, each of which has a distinct impact on the response variable, R. Specifically, an increase in factor A by one unit is associated with a decrease in R by 0.7375 units, assuming all other factors remain unchanged. Similarly, if factor B increases by one unit, the response R is anticipated to decrease by 0.1 units, given that all other factors are constant. Finally, an increase in factor C by one unit is expected to result in a decrease in R by 0.4605 units, with all other factors held constant.

Actual equation:

(2) Porosity = −233.287 + 0.48075 × (A) + 4.075 × (B) - 4.8325 × (C) - 0.002 × (A × B) + 0.0045 × (A × C) + 0.04 × (B × C) - 0.000265 × (A^2^) - 0.0515 × (B^2^) - 0.0265 × (C^2^)

## 3 Result and discussion

### 3.1 As sprayed coatings

**Fig. 3:**
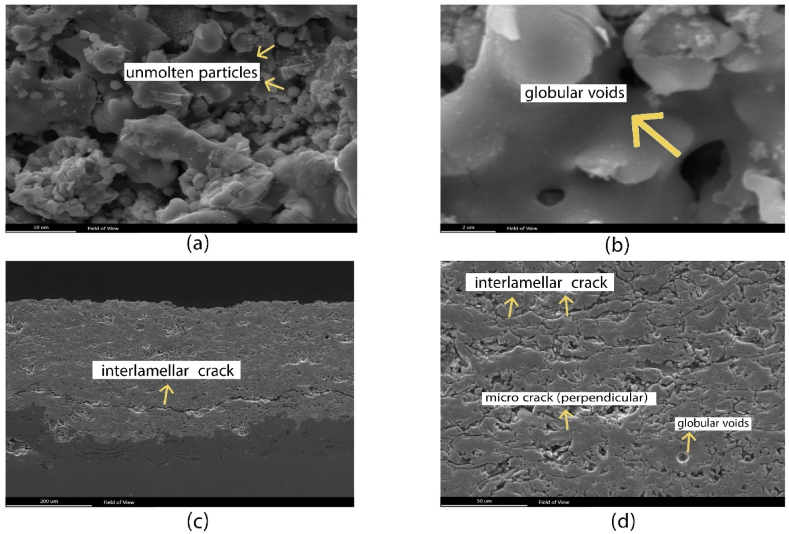
(a, b) FE-SEM images showcasing the surface of the as-sprayed (as-APS) coatings. (c, d) Cross-sectional views of the as-APS coatings, highlighting various defects.

Surface morphology and cross-sectional microstructure of the as-plasma-sprayed coating using FE-SEM are shown in Fig. 3. Plasma spray coatings exhibit predominant defects such as voids, unmolten particles, microcracks, macrocracks, and unsplashed particles. Previous research investigations [[12], [13], [14], [15]] have categorized voids in plasma spray coatings into globular voids, perpendicular cracks, and interlamellar cracks. The spherical morphology of unmolten particles, depicted in Fig. 3 (a), is a result of chemical reactions and atmospheric gases during the APS technique [17]. Vertical cracks or microcracks, characterized by elongated voids formed perpendicular to the substrate due to the coating growth, are apparent in Fig. 3 (d). Under additional stress or exposure to severe environments, these microcracks can serve as initiation sites for the propagation of larger cracks [18]. Fig. 3 (c) illustrates a cross-section area of the as-APSed coating, revealing an extended interlamellar crack. The presence of interlamellar cracks in the coating can compromise its structural integrity, leading to decreased performance. Based on the analysis, the average porosity of the coatings was determined to be 20 %. Porosity is primarily generated during the rapid solidification process due to the entrapment of air sacks and solidification shrinkage. Unmolten particles are a common issue in plasma spray coatings and can lead to the formation of globular porosity [31]. These globular defects are homogeneously disseminated throughout the coating and pose a high risk of deteriorating the coating properties. Fig. 3 (c) presents the average thickness of the as-APS top coating, which is found to be around 340 μm. Fig. 3 (d) reveals the existence of spherical voids with diameters varying between 4 and 10 μm.With the characteristics of the as-sprayed coating discussed, attention can now be turned to the alterations in these properties brought about by the process of consolidation by SPS.

### 3.2 Consolidation of the coatings via spark plasma sintering

As depicted in Table 3, the coatings demonstrated a reduction in interlamellar cracks following the SPS treatment conducted at temperatures of 900 and 950, and under a pressure of 30 MPa. However, both open and closed porosities were still observed. Generally, as the temperature increased, the level of porosity decreased. Interestingly, the highest level of porosity was observed when the pressure was at a mid-point of 25 MPa. The data indicates that a high temperature, when coupled with either low or high pressure, leads to a decrease in porosity. To illustrate, experiments 3 and 9, which were conducted at 950 °C and pressures of 30Mpa and 20Mpa respectively, demonstrated a 13 % decrease in porosity following the SPS process. As the holding time increased, there was a noticeable decrease in porosity. For example, during run 6, when the holding time was set to 15 min, there was a 16 % reduction in the level of porosity. SPS process, which simultaneously applies pressure and temperature, has demonstrated its effectiveness in reducing the thickness of coatings. This process involves the concurrent application of high temperature and pressure, leading to the densification of the material and a subsequent decrease in porosity. As a result, the coating thickness is reduced while maintaining its structural integrity. Following the exploration of the consolidation process, it becomes crucial to assess the accuracy of the model in predicting these changes.

### 3.3 Assessing model adequacy

The ANOVA table and related statistics provide valuable insights into the significance of model terms, goodness of fit, precision of predictions, and reliability of the regression equation obtained from RSM [32]. These findings are important in understanding the relationship between process parameters and porosity levels, optimizing process conditions, and ensuring consistent and reliable product quality [33]. In this analysis, the significant model terms identified in the ANOVA table (Table 4) provide valuable insights into the factors that have a significant impact on porosity. The interactions AB, BC, and AC are found to be significant, indicating that the combined effects of these factors on porosity cannot be ignored. This information can be used to optimize process parameters and achieve desired levels of porosity in the final product. The high Model F-value of 301.02 further confirms the overall significance of the model, indicating that the regression equation derived from the RSM analysis is a reliable predictor of porosity levels.

**Table 4:** Analysis of variance (ANOVA) results and fit statistical parameters of the model equation.

Source	Sum of Squares	df	Mean Square	F-value	p-value	
Model	41.77	9	4.64	301.02	<0.0001	significant
A-A	2.53	1	2.53	164.09	<0.0001	
B–B	0.0465	1	0.0465	3.02	0.1331	
C–C	1.21	1	1.21	78.78	0.0001	
AB	1.0000	1	1.0000	64.86	0.0002	
AC	5.06	1	5.06	328.38	<0.0001	
BC	4.00	1	4.00	259.46	<0.0001	
A^2^	1.76	1	1.76	113.88	<0.0001	
B^2^	6.63	1	6.63	430.09	<0.0001	
C^2^	1.76	1	1.76	113.88	<0.0001	
Residual	0.0925	6	0.0154			
Lack of Fit	0.0625	3	0.0208	2.08	0.2810	Not significant
Pure Error	0.0300	3	0.0100			
Cor Total	41.86	15				
Std. Dev.	**Mean**	**C.V. %**	**R**^**2**^	**Adjusted R**^**2**^	**Predicted R**^**2**^	**Adeq Precision**
**0.1242**	7.64	1.62	0.9978	0.9945	0.9748	65.5816

Furthermore, the Lack of Fit F-value of 2.08 suggests that the Lack of Fit is not statistically significant compared to the pure error, indicating that the model fits the data well. The Fit statistics in Table (6) provide additional evidence of the model's accuracy and reliability. The high Predicted coefficient of determination (predicted-R^2^) of 0.9748 indicates that the model can explain a significant portion of the variability in the response variable. The Adjusted R^2^ of 0.9945 further supports the model's goodness of fit, as it accounts for the number of model terms and adjusts the R^2^ value accordingly.

Additionally, the model's ability to make precise and accurate predictions of porosity levels is indicated by an Adeq Precision ratio (signal-to-noise ratio) of 65. 582. The coefficient of the variant (C.V%) is also in good agreement with the mean sum of squares, indicating that the residual variability in the data is minimal. The low C.V% value suggests that the method used for porosity measurement is reliable and consistent, with a small percentage of variability relative to the mean of the response variable. Additionally, the standard deviation (Std. Dev) of 0.1242 for the porosity measurement model reflects the precision of the model in predicting porosity levels. To further validate the accuracy of the model, normal probability plots will be employed, serving as a powerful tool for visualizing and understanding the distribution of residuals.

### 3.4 The normal probability plots

**Fig. 4:**
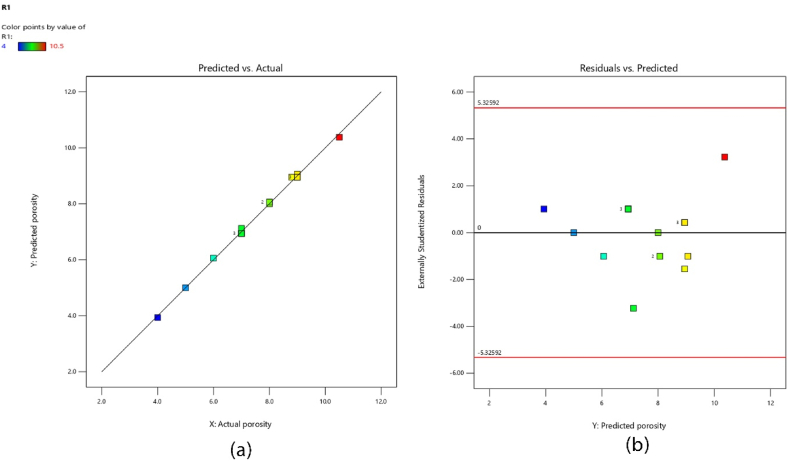
diagnosis plots of (a) The predicted versus actual values (b)externally studentized residuals.

The predicted versus actual plot is a valuable tool in analyzing the performance of a predictive model [34]. It compares the predicted values of the response variable with the actual observed values. Fig. 4(a) demonstrates the accuracy of the model by showcasing how closely the data points align with the ideal diagonal line, symbolizing a perfect prediction. Certainly. Fig. 4(b) is a visual representation that demonstrates the relationship between the predicted values obtained from the model and their corresponding externally studentized residuals. In the context of this study, Externally Studentized residuals were utilized for regression analysis. This approach was selected due to the varying leverages across different runs in the design, which resulted in differing standard errors for the residuals. The use of raw residuals for verifying regression assumptions was found to be unsuitable, as they belong to different populations. To address this, the residuals were Studentized, effectively mapping all normal distributions onto a single standard normal distribution. Upon examination of the scatter plot of these Studentized residuals, a random dispersion of data points was observed, with no discernible pattern. This randomness in the residuals is a positive indicator, suggesting that the assumptions of the regression model are likely being met. This finding has significant implications for the robustness and reliability of the model. With an understanding of the distribution of residuals established, the focus will shift to the perturbation plot to comprehend the interactions between variables and their impact on the response.

### 3.5 Perturbation plot and interactions

**Fig. 5:**
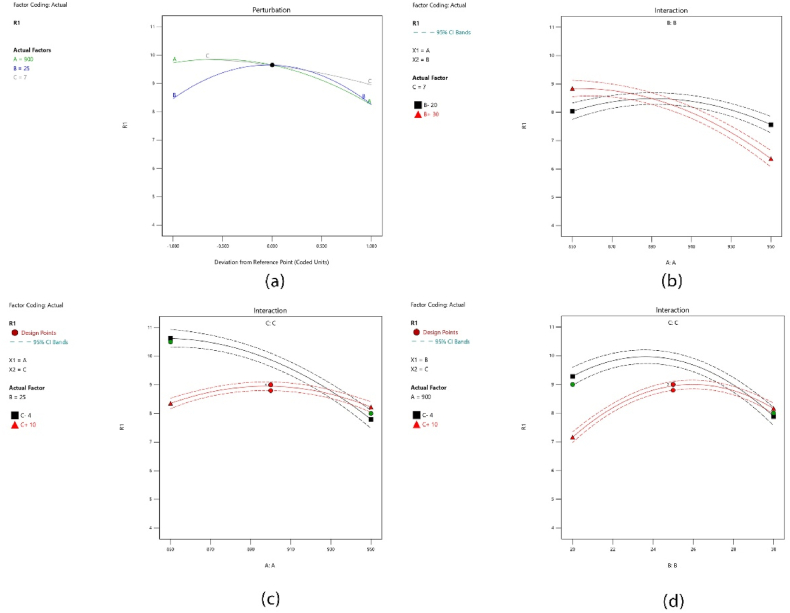
(a) perturbation plot and (b, c, d) interaction effects of AB, AC, and BC, respectively.

Fig. 5 illustrates the perturbation plot and interactions. In Fig. 5(a), the perturbation plot showcases variations in the response variable (porosity) as individual factors deviate from their midpoints, set as the reference points in the design-expert software. The porosity level is plotted by altering one factor at a time within its range while keeping all other factors constant. The plot provides insight into how each factor affects the response variable. Moreover, utilizing F-values allows for the identification of predominant factors that exert significant effects on the responses, encompassing both major and minor influences. The evaluation suggests that temperature, holding time, and pressure emerge as the primary parameters directly impacting porosity. Fig. 5(b), (c), and (d) display the interaction plots that depict the joint influence of factors A, B, and C on the response variable. In Fig. 5 (b), the interaction between temperature and pressure (AB) is illustrated. When keeping the pressure constant at 20 MPa (represented by black squares), a slight decrease in porosity level is observed as the temperature increases. However, when maintaining the pressure at 30 MPa (indicated by red triangles), the porosity level exhibits a more significant decrease with increasing temperature. Fig. 5(c) illustrates the interaction between temperature and holding time (AC). The plot highlights that as temperature rises, the porosity decreases notably, particularly when maintaining a constant holding time of 4 min. This suggests that temperature has a stronger influence on porosity when the holding time is held at 4 min. Fig. 5 (d) depicts the interaction between pressure and holding time (BC) and its impact on porosity variations. When the holding time is held constant at 10 min, the plot indicates that the porosity level increases as the pressure rises. However, when the holding time is lower (4 min), the plot shows that the porosity level initially increases with pressure, but then decreases smoothly. This suggests that the effect of pressure on porosity depends on the holding time, with different patterns observed at different holding time levels. Having examined the interactions between variables, relationships can now be visualized more intuitively using contour and 3D surface graphs.

### 3.6 Contour and 3D surface graphs

**Fig. 6:**
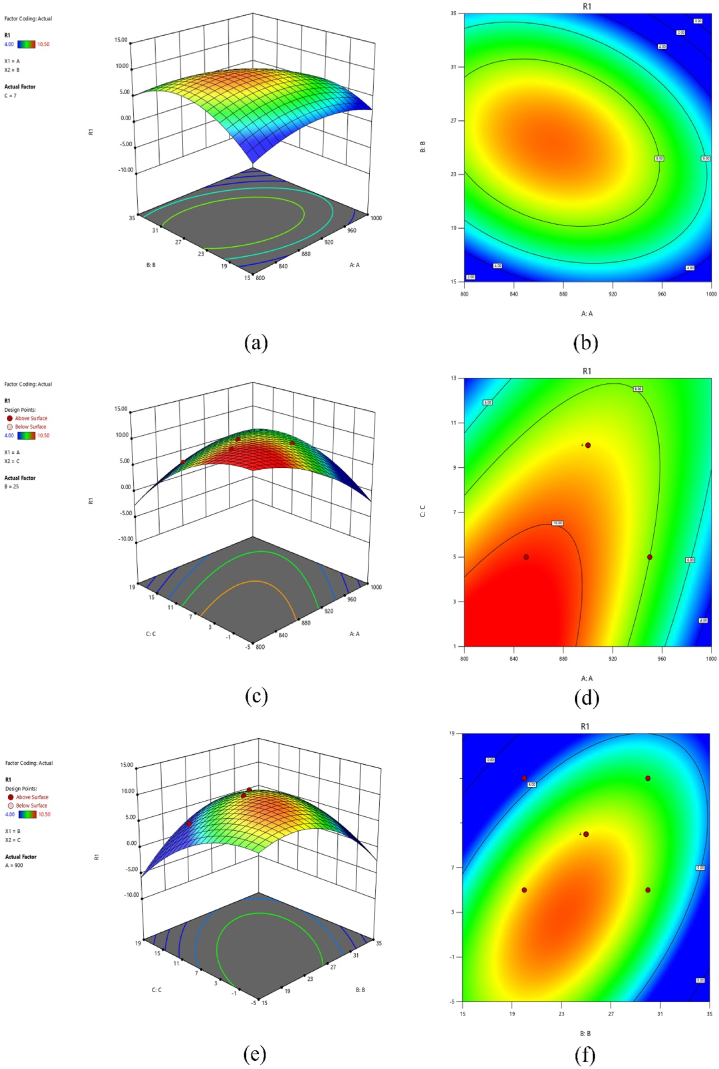
Contour Plots and 3D Surface Graphs, Visualizing the Effects of interaction Parameters on Porosity Levels. (a, b) temperature and pressure. (c, d) temperature and holding time. (e, f) pressure and holding time.

In response surface methodology, response contour plots and 3D surface graphs are essential tools for understanding the effects of variables on a response within a given experimental domain. In Fig. 6, these graphical representations are used to illustrate the impact of three variables on the porosity levels. Table 3 provides details of the experimental design and the corresponding results for the conducted experiments. The table presents SEM images of coatings obtained after undergoing the SPS process for different experimental runs. The observations include porosity level and coating thickness. Fig. 6 (a, b) show the interaction effect of temperature and pressure on porosity, revealing that the highest porosity levels are observed at temperatures below 930 °C and pressures above 22 MPa. This implies that when lower temperatures are combined with higher pressures, it may lead to higher porosity levels, as evident from the observations in Table 3, specifically in runs 3 and 6. The blue regions illustrated on the 3D graph represent areas characterized by the lowest observed porosity levels. These regions exhibit a correlation with higher temperatures and lower pressures, which is consistent with the findings reported in a previous study [9]. This alignment highlights a consistent pattern in the relationship between temperature, pressure, and porosity levels within the fabricated coatings. The findings of this study [35] validate that the lowest porosity levels were achieved at elevated temperatures and intermediate pressures. In Fig. 6 (c, d), the contour plot demonstrates the interaction effect of temperature and holding time (AC). It reveals that lower porosity levels can be achieved by using higher temperatures (e.g., 950 °C) and shorter holding times (e.g., 4 min). When coatings are exposed to high temperatures for shorter durations, the particles may undergo rapid bonding and rearrangement, resulting in a tighter and more compact structure with reduced porosity. Khor et al. [31] found that the application of elevated temperature (1500 °C) and a holding time of 3 min during the SPS process resulted in the coatings exhibiting the lowest porosity levels. Similarly, the combined effect of pressure and holding time is depicted in Fig. 6 (e, f). The contour plot shows that lower porosity levels can be achieved at lower holding times and higher pressures. The red zone in the contour plot highlights the area with the highest porosity levels, where pressure varies from 22 to 26 MPa and holding time ranges from 4 to 7 min approximately. This suggests that a combination of higher pressures and shorter holding times may result in increased porosity levels. With a comprehensive understanding of variable interactions and their graphical representations established, the process can now be optimized for the most desirable outcomes.

### 3.7 Optimization process

**Fig. 7:**
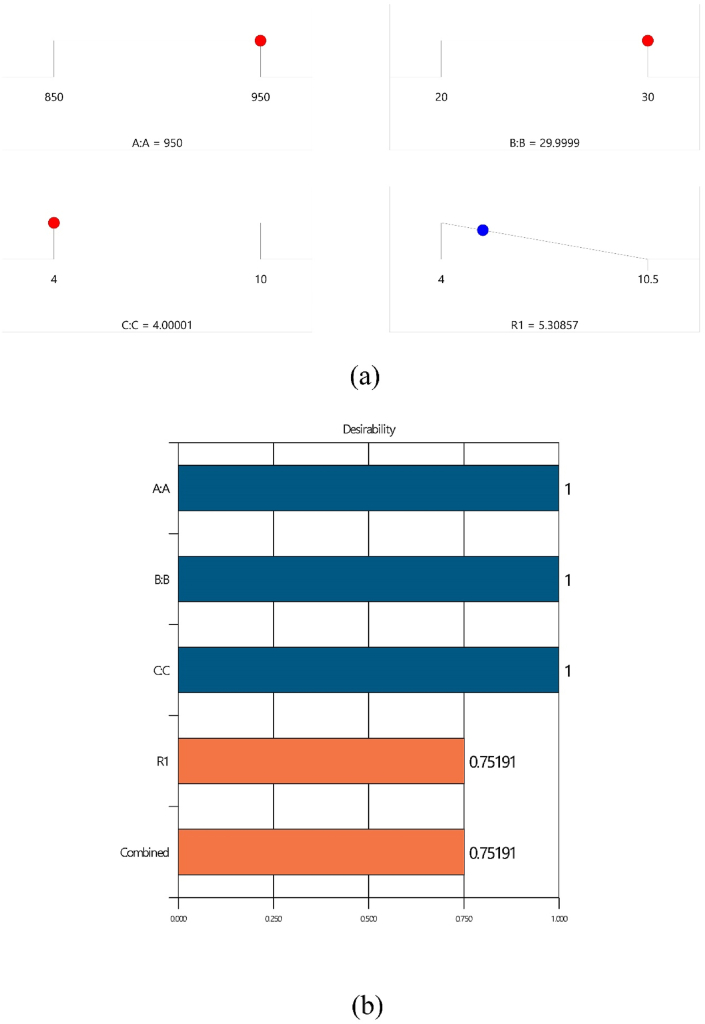
(a) ramp, (b)bar plot on desirability of the Independent and dependent variables.

The results of the experiment are presented with a 95 % confidence interval, indicating that if the experiment were repeated, the actual population mean would fall within this interval 95 % of the time. Fig. 7(a) displays a ramp plot where three independent variables - temperature, pressure, and holding time - are set to specific values of 950, 30, and 4, respectively. The desired output, porosity, is set to 5. The ramp plot aids in identifying the optimal combination of input variables that yield the desired response. The red dots on the graph represent the optimal state of the input variables, while the blue dot shows the achievement of the goal (low porosity).

The Desirability Function (DF), a multi-response optimization technique, was employed to find the optimal point that results in the lowest porosity level. The DF seeks a combination of input variables that produce the best response. The input variables were selected within their specific range with the aim to minimize the response. In this method, the response is converted into a desirability value, a scale-free value ranging from 0 to 1. Optimal conditions are achieved when desirability is maximized.

Table 5 lists various combinations of input variables that maximize the desirability value, providing predicted response values and desirability for each combination. This table offers valuable insights for identifying the most favorable parameter settings that yield the desired output. Fig. 7(b) depicts the desirability of input variables and response separately, as well as the combined desirability of all input variables and response. The calculated combined desirability value of 0.75191 in this experiment suggests a favorable condition for numerical optimization.

**Table 5:** Optimized conditions.

Number	A	B	C	R1	Desirability	Desirability (w/o Intervals)	
1	**950.000**	**30.000**	**4.000**	**5.309**	**0.752**	**0.799**	**Selected**
2	949.387	30.000	4.000	5.348	0.746	0.793	
3	950.000	29.940	4.000	5.353	0.746	0.792	
4	948.934	30.000	4.000	5.377	0.742	0.788	
5	950.000	30.000	4.183	5.386	0.741	0.787	
6	950.000	29.866	4.000	5.409	0.738	0.783	
7	950.000	30.000	4.268	5.422	0.737	0.781	
8	950.000	29.800	4.000	5.458	0.731	0.776	
9	949.979	30.000	4.645	5.576	0.715	0.758	
10	949.517	29.683	4.000	5.573	0.715	0.758	
11	950.000	29.632	4.000	5.580	0.714	0.757	
12	947.022	29.881	4.000	5.588	0.713	0.756	
13	943.657	29.999	4.000	5.710	0.695	0.737	
14	942.491	30.000	4.000	5.780	0.685	0.726	
15	950.000	29.341	4.011	5.788	0.684	0.725	
16	950.000	30.000	5.424	5.868	0.675	0.713	
17	950.000	29.233	4.000	5.858	0.674	0.714	
18	950.000	30.000	5.585	5.924	0.667	0.704	

## 4 Conclusion

In conclusion, this study successfully achieved the fabrication of dense SPSed CeO_2_/GNP coatings as a post-treatment approach to reduce porosity, interlamellar cracks, vertical cracks, and voids in APS coatings.

• The CeO_2_/GNP composite coating was successfully fabricated on stainless-steel substrate using a three-step process involving high-energy ball milling, spray drying, and air plasma spray.
• The further densification of as-APS coatings was carried out using spark plasma sintering (SPS) as a post-treatment approach. By optimizing the SPS process parameters using the Response Surface Methodology and considering the simultaneous combination effect of temperature, pressure, and holding time, the porosity level was reduced from 20 % in APS coatings to 5 % in the optimized SPSed coatings.
• The presence of various defects such as voids and cracks (interlamellar and micro) in APS coatings was confirmed, and the densified SPSed coatings exhibited improved versatility and reduced defects.
• The combined impact of process parameters shows that higher pressures have a greater influence on porosity when the temperature is also high, rather than low pressures. Additionally, temperature has a stronger effect on porosity at a holding time of 4 min compared to other values. The interaction between pressure and holding time reveals that at 10 min holding time, increasing pressure increases porosity, while at 4 min holding time, porosity initially increases with pressure and then decreases. Proper consideration of these interrelated process parameters is essential for effective porosity control in SPS.
• A regression equation for porosity based on three independent parameters was developed, which could assist in accurate porosity prediction.
• These findings highlight the effectiveness of the SPS post-treatment approach in achieving denser coatings with reduced porosity and improved quality. Further research and application of these optimized SPS parameters can lead to enhanced performance and durability of APS coatings in various industrial applications.
• Through the optimization process, the constraints for each parameter have been established. The temperature is set at 950 °C, the pressure at 30 MPA, and the holding time at 4 min.

## Data availability

Data will be made available on request.

Data associated with this study has not been deposited into a publicly available repository.

## CRediT authorship contribution statement

**Ali Jounaki:** Writing – review & editing, Writing – original draft, Resources, Project administration, Methodology, Investigation, Formal analysis, Data curation, Conceptualization. **Seyedeh Zahra Anvari:** Writing – review & editing, Conceptualization.

## Declaration of Generative AI and AI-assisted technologies in the writing process

During the preparation of this work the authors used Open AI chat GPT in order to improve readability and language. After using this tool, the authors reviewed and edited the content as needed and take full responsibility for the content of the publication.

## Declaration of competing interest

The authors declare that they have no known competing financial interests or personal relationships that could have appeared to influence the work reported in this paper.
